# Bone marrow edema in the knee in osteoarthrosis and association with total knee arthroplasty within a three-year follow-up

**DOI:** 10.1007/s00256-008-0504-x

**Published:** 2008-07-01

**Authors:** Courtney Scher, Joseph Craig, Fred Nelson

**Affiliations:** 1 Henry Ford Macomb Hospital, Michigan State University Osteopathic Radiology Residency, Warren Campus, 13355 West Ten Mile Road, Warren, MI 48089 USA; 2 Henry Ford Hospital, Department of Radiology, 2799 West Grand Blvd., Detroit, MI 48202 USA; 3 Henry Ford Hospital, Department of Orthopaedic Surgery, 2799 West Grand Blvd., Detroit, MI 48202 USA

**Keywords:** Osteoarthrosis, Bone marrow edema, Total knee arthroplasty

## Abstract

**Objective:**

The purpose of this study was to determine if a correlation exists between magnetic resonance imaging (MRI) findings of bone marrow edema (BME) in osteoarthrosis (OA) of the knee joint and need for total knee arthroplasty (TKA) within a follow-up period of 3 years.

**Materials and methods:**

The entire database of knee MR studies over a 3-year period was used to select individuals with knee OA. A chart review was conducted to identify and include only those who had a 3-year follow-up appointment from the time of the initial MR study. There were 25 patients in the OA-only group (four men and 21 women; age range, 28–75; average age, 49.3 years). The OA and BME group had 48 patients (23 men and 25 women; average age, 55.5 years). The MRs were reviewed and interpreted by a musculoskeletal radiologist and were classified into one of four patterns of BME: none, focal, global, or cystic pattern. Meniscal tear and degree of cartilage loss were also assessed.

**Results:**

Subjects who had BME of any pattern type were 8.95 times as likely to progress rapidly to a TKA when compared to subjects with no BME (*p* = 0.016). Subjects with a global pattern of BME were 5.45 times as likely to have a TKA compared to subjects with focal, cyst, or no BME (*p* < 0.05). Subjects with a global edema pattern were 13.04 times as likely to have a TKA than subjects with no marrow edema in the knee (*p* < 0.01). There was no correlation of TKA with meniscal tear or cartilage loss. The group of subjects who had a TKA were 12.6 years older than those who did not have a TKA (*p* < 0.001). However, the BME results were still significant after accounting for the age difference.

**Conclusion:**

Our classification of patterns into global, focal, cystic, and absence of BME is an attempt to further define edema in osteoarthrosis and how it relates to clinical progression. Patients with BME and OA have an increased risk of TKA as opposed to OA and no marrow edema. The BME pattern with the worst prognosis for the knee is the global pattern.

## Introduction

Osteoarthrosis (OA) is the most common cause of disability among the elderly population [1, 2]. OA is a condition where articular cartilage cannot maintain homeostasis in response to the forces acting on it. When homeostasis breaks down, there is a wide range of possible biologic responses based on the genetic background of the individual [3]. These responses may be anabolic or catabolic and involve cartilage, bone, and synovium. The exact pathogenesis of OA and the cause of the pain produced are unclear. OA is classically associated with degradation of the hyaline cartilage around the joint. However, cartilage contains no pain fibers. Other proposed sources of pain production in OA include periarticular structures and the underlying bone [3].

Several recent studies have examined bone marrow edema (BME) lesions and their association with OA [1, 4–10]. BME has been defined as an area of low signal intensity on T1-weighted images, associated with intermediate or high signal intensity findings on T2-weighted images. The literature suggests that there are two distinct types of BME. The first type occurs traumatically with injury to a joint and tends to resolve over a period of weeks to months [11–14]. This type of BME does not appear to have any long-term implications [15, 16]. The second type of BME occurs without trauma and may be associated with rapidly progressing osteoarthrosis. Zanetti et al. [9] suggest a possible link between atraumatic BME and increased pain in OA. Felson et al. [7] have suggested a possible link between BME lesions on magnetic resonance imaging (MRI) and progression of OA findings on MRI over time. Hunter et al. found that bone marrow lesions that enlarge with time are associated with more cartilage loss, compared with bone marrow lesions that stay the same size over time [17].

Despite the recent literature on BME, the implication of these findings on MRI and how they relate to clinical outcomes is unknown. Most studies have used the subjective complaint of pain as the only clinical correlate. There has been no measure of an objective clinical outcome related to the finding of BME. The purpose of this study was to determine if a correlation exists between MRI findings of BME of the knee joint and the incidence of total knee arthroplasty (TKA) within a follow-up period of 3 years.

## Materials and methods

The entire database of knee MRI studies from 1995–1997 (over 4,000 studies) conducted at a large urban hospital system was used to select individuals with knee osteoarthrosis (OA). The study was reviewed and approved by the hospital’s institutional review board. An initial random search was conducted within this database to identify two distinct groups of patients. The first group had MRI reports containing the phrase “knee osteoarthrosis/osteoarthritis,” and the second group consisted of those containing the phrases “knee osteoarthrosis/osteoarthritis” and “bone edema.” The search for “osteoarthrosis/osteoarthritis” yielded 235 cases for review. The search for “osteoarthrosis/osteoarthritis” and “bone edema” yielded 146 cases for review. After these two initial groups were identified, a chart review was conducted on all 381 patients to identify and include only those patients who had at least a 3-year clinical follow-up appointment from the time of the MRI study. Subjects were also excluded if the reason for referral to MRI was post-traumatic or post-surgical. An initial review of the images was completed by an experienced musculoskeletal radiologist to exclude any subjects with evidence of recent post-surgical or post-traumatic changes not mentioned in the report. After this chart review and initial radiology review process, there were 38 patients in the OA-only group and 35 in the OA with BME group.

The same musculoskeletal radiologist who provided the initial review was blinded to the original interpretation of the MRI studies and the patient outcome, and reviewed all 73 studies. The radiologist was aware of the study hypothesis at the time of interpretation. The radiologist assessed each knee for the presence or absence of BME and assigned each subject into either the BME group or the no-BME group for further evaluation. After this review of the images, there were a total of 25 patients with OA only and 48 with OA and BME.

The OA-only group consisted of four males and 21 females, with an age range of 28–75 years, and an average age of 49.3 years. Conventional radiographs consisting of anteroposterior, lateral and sunrise views were available for review with 13 of the 25 patients, 52% of the group.

The OA and BME group consisted of 23 men and 25 women, with an age range of 35–82 years and an average age of 53.5 years. Conventional radiographs, again consisting of anteroposterior, lateral, and sunrise views, were available for review with 33 of the 48 patients, 68.75% of the group.

The conventional radiographs, when available, were reviewed by the musculoskeletal radiologist. Radiographic evidence of OA on the radiographs was assessed using the Kellgren–Lawrence scale [18] as follows:

(0) None
(1) Doubtful
(2) Minimal
(3) Moderate
(4) Severe

According to this scale, evidence of OA includes (1) formation of osteophytes on joint margins or on the tibial spine, (2) periarticular ossicles, (3) narrowing of joint cartilage associated with sclerosis of the subchondral bone, (4) small pseudocystic areas with sclerotic walls situated usually in the subchondral bone, and (5) altered shape of the bone ends [18].

All patients had dedicated knee MR scans either on a GE 1.5-T magnet (23 in the OA-only group or 92% and 42 subjects in the OA with edema group or 87.5%) or a 1.0-T magnet (8% of the OA-only group, two subjects, and 12.5% of the OA with edema group, six patients).

MRI films were reviewed and interpreted by the musculoskeletal radiologist for all patients in the study. For each patient, the sagittal proton density sequences (TR range, 2,666–4,100; TE range, 14–33; 44 conventional spin echo studies [ET = 0], 29 fast spin echo studies [ET = 4–8], 3- to 5-mm section thickness, 16 × 16–18 × 18 field of view [FOV], 256 × 128–256 image matrix, bandwidth range 16–32), coronal proton density sequences (TR range, 2,316–3,950; TE range, 14–33; 44 conventional spin echo studies [ET = 0]; 29 fast spin echo studies[ET = 4–8]; 3-mm section thickness; 16 × 16–18 × 18 FOV; 256 × 128–256 image matrix; bandwidth range, 16–32), and coronal fat suppressed T2-weighted images (TR range, 2,400–3,200; TE range, 56–80; 3- to 5-mm section thickness; 16 × 16–18 × 18 FOV; 256 × 128–256 image matrix; bandwidth range, 15.6–16) were reviewed. Axial fat suppressed T2-weighted images (TR range, 3,000–3,600; TE range, 56–76; 1.5- to 10-mm section thickness; 16 × 16–20 × 20 FOV; 256 × 128–256; bandwidth range, 15.6–16) were available and reviewed for 69 of the 73 knees. For the four subjects who had no axial T2 imaging available, BME in the patellofemoral joint was not assessed.

The medial, lateral, and patello-femoral compartments of the knee joint were assessed. Evidence of OA on MRI was assessed using a modified Noyes arthroscopic classification [19] as follows:

- Normal
- Internal changes only
- 1–49% loss of articular cartilage
- 50–99% loss of articular cartilage
- 100% loss of cartilage with subchondral cortex intact
- 100% loss of cartilage with ulcerated subchondral cortex

The most severe grade cartilage lesion within a compartment was utilized for the grading of that compartment. Also, the highest grade cartilage lesion on either side (femoral or tibial) of a compartment was used.

**Fig. 1:**
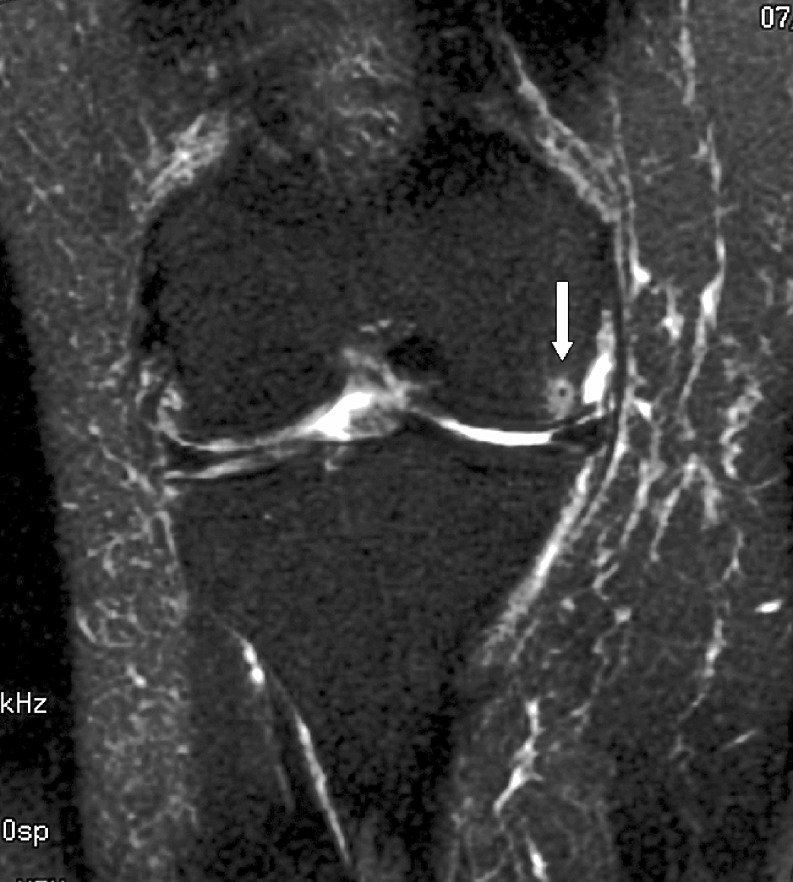
Focal edema with a total knee joint replacement in a 51-year-old female patient. The radiograph demonstrated moderate loss of cartilage joint space medially and spurring of the tibial spines. Coronal fast fat suppressed T2-weighted image (TR 3000/TE 76/256 × 192) shows the loss of hyaline cartilage and a small focal area of edema in the medial aspect of the medial femoral condyle (*arrow*)

**Fig. 2:**
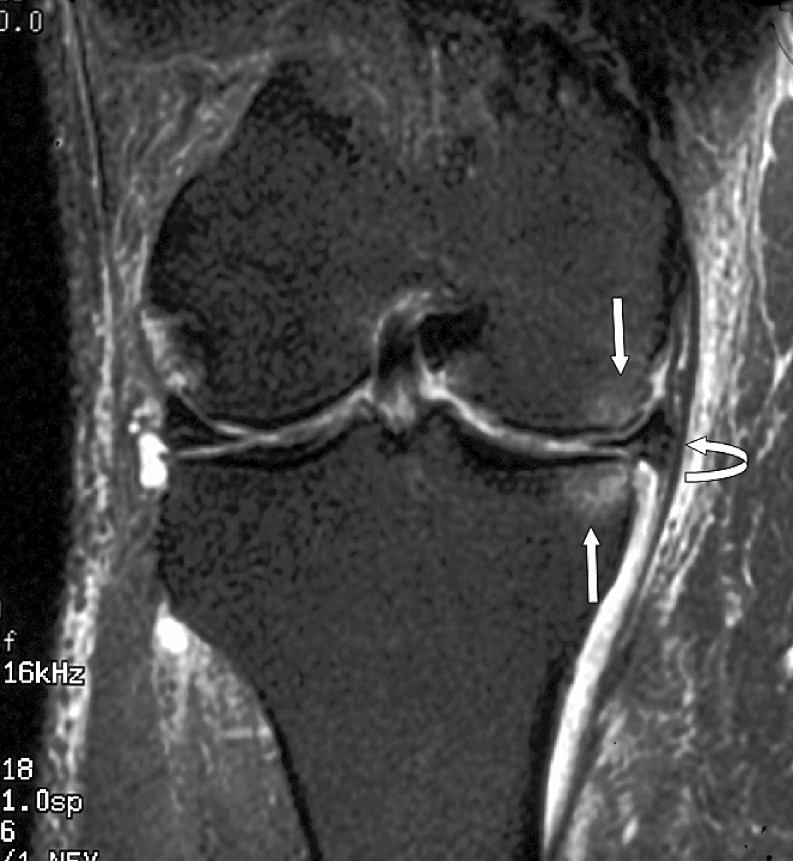
Focal edema, no total knee joint replacement, in a 58-year-old male patient. Coronal fast fat suppressed T2-weighted image (TR 3000/TE 56/256 × 192) shows extrusion of the medial meniscus (*curved arrow*) and small adjacent areas of focal marrow edema (*straight arrows*). The medial meniscus has a degenerative horizontal cleavage tear approximating the tibial surface. Note also the moderate loss of hyaline cartilage

**Fig. 3:**
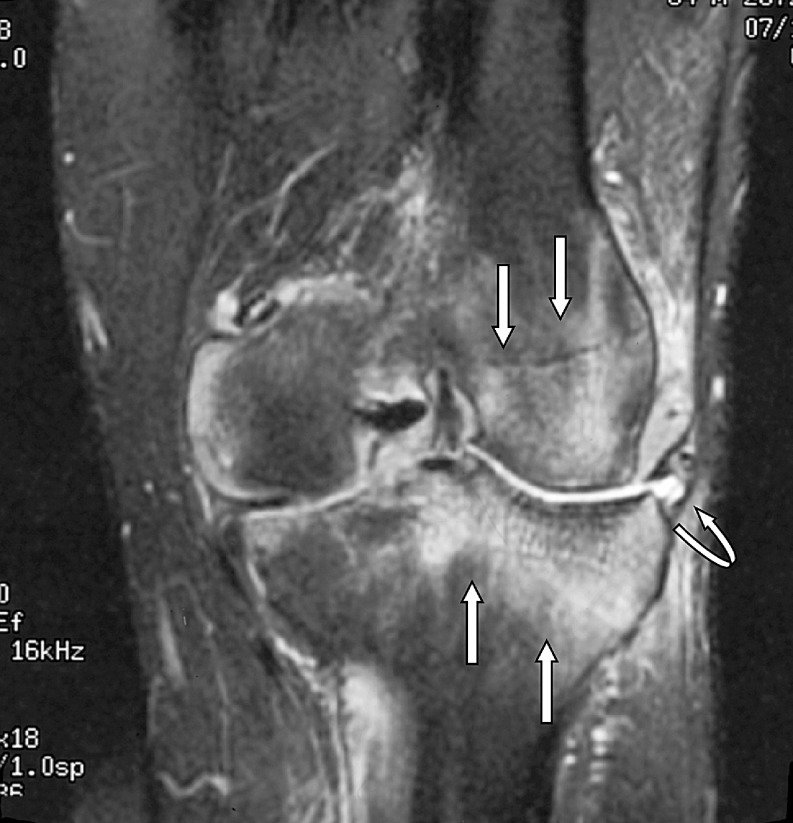
Global edema with a total knee joint replacement in a 65-year-old male patient. The radiograph demonstrated absence of joint space in the medial and lateral compartments, and irregularity of the subchondral bone plate of the lateral femoral condyle. Coronal fast fat-suppressed T2-weighted image (TR 3000/TE 72/256 × 192) through the knee shows extensive edema in the lateral femoral and tibial condyles, extending into the metaphyses (*straight arrows*). Note also the small extruded body of the degenerative and torn lateral meniscus (*curved arrow*)

**Fig. 4:**
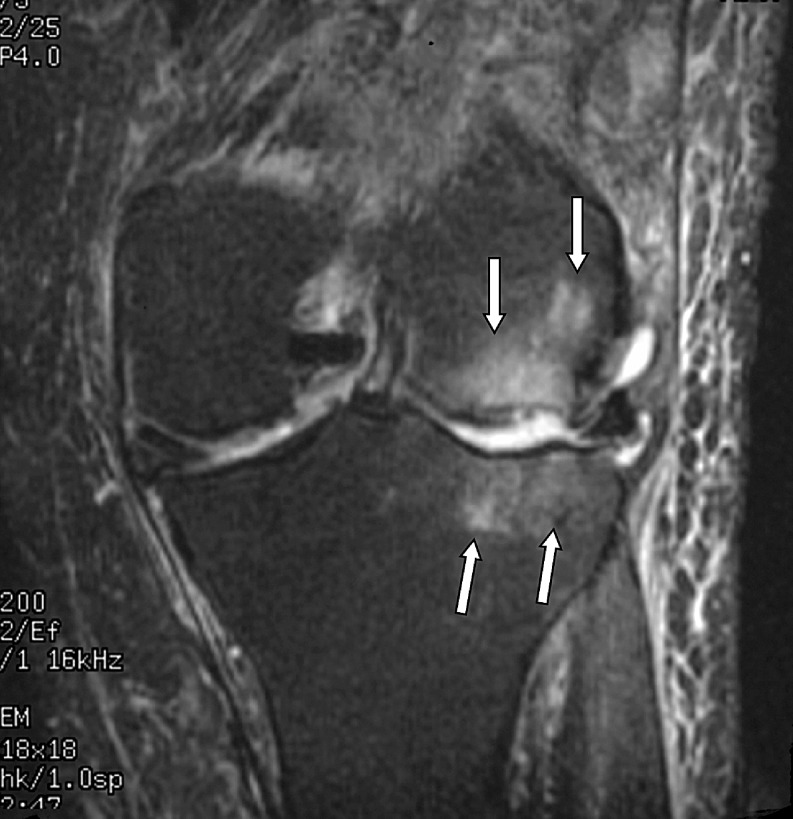
Global edema with a total knee joint replacement in a 72-year-old male patient. The radiograph demonstrated loss of cartilage joint space within the lateral compartment. Coronal fast fat-suppressed T2-weighted image (TR 3200/TE 72/256 × 192) shows extensive edema in the femoral and tibial condyles (*arrows*)

**Fig. 5:**
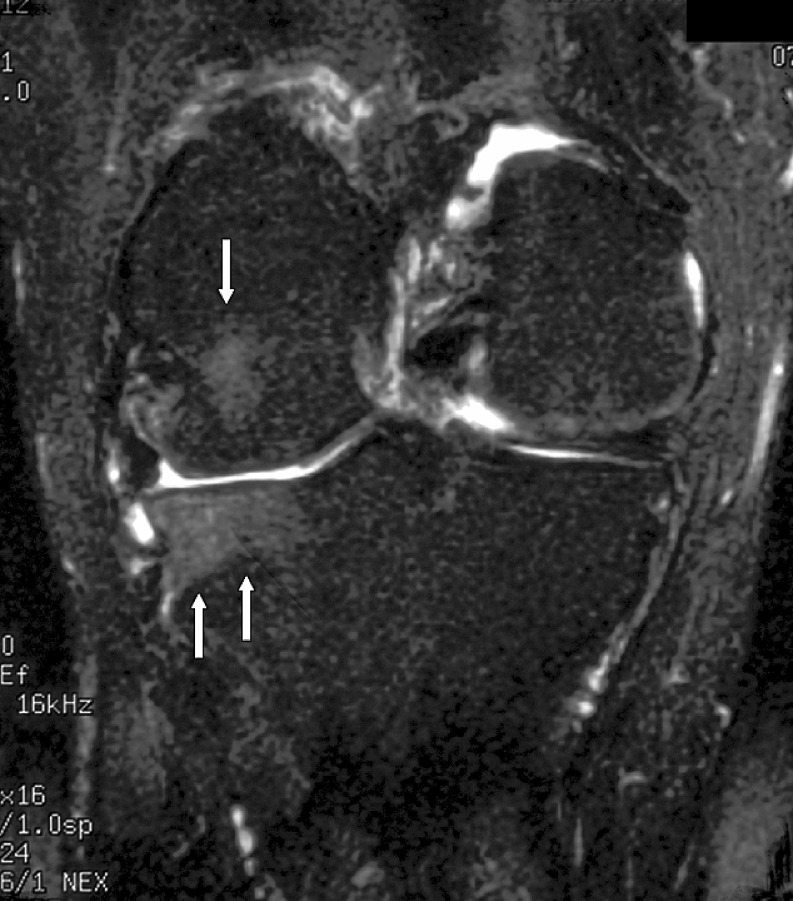
Global edema, no total knee joint replacement, in a 58-year-old male patient. Coronal fast fat suppressed T2-weighted image (TR 3000/TE 76/256 × 256) through the knee shows extensive edema in the lateral tibial condyle and edema in some of the lateral femoral condyle (*arrows*). Note also the almost complete absence of hyaline cartilage

**Fig. 6:**
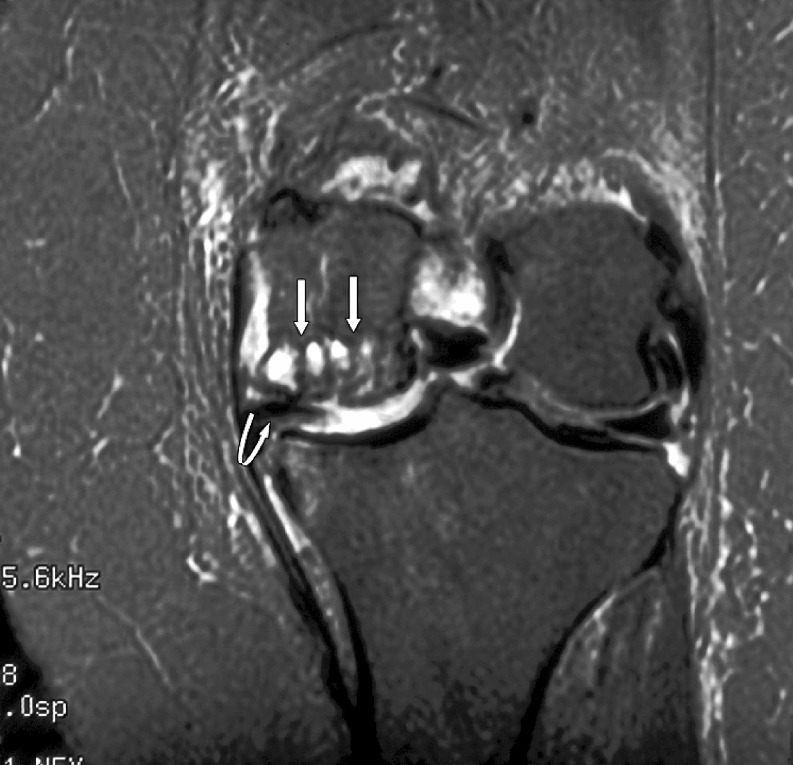
Cystic pattern with a total knee joint replacement in an 82-year-old female patient. The radiograph demonstrated subchondral lucency consistent with a geode and irregularity of the adjacent subchondral bone plate. Coronal fast fat-suppressed T2-weighted image (TR 3000/TE 56/256 × 192) shows complete loss of hyaline cartilage on the medial femoral condyle and adjacent moderate subchondral cystic areas consistent with geodes, and the adjacent bone marrow edema (*arrows*). Note also the extruded body of the medial meniscus containing a degenerative horizontal cleavage tear (*curved arrow*)

The radiologist then assessed each knee MRI for the presence or absence of BME. BME was defined as an area of intermediate or high-signal intensity findings on T2-weighted images. All patients in the BME group were then further classified into three separate, groups based on patterns of BME. Type I was termed “focal” and contained a small area of edema adjacent to a cartilage defect (Figs. 1 and 2). Type II was termed “global” pattern with marrow edema occupying most or all of a femoral or tibial condyle (Figs. 3, 4, and 5), and type III was termed “cystic” and was associated with a subchondral geode with a small area of BME (Fig. 6). The worst area of edema in a compartment was used in the classification. The presence or absence of meniscal tear was also noted.

Generalized estimating equations (GEE) were used to determine which factors were related to receiving a TKA. There were eight subjects who had bilateral knee MRIs. GEE takes into account the correlation within an individual who had both knees involved, whereas a general linear model assumes that all observations are independent and does not allow for multiple observations from one individual. A multivariable logistic regression model using generalized estimation was also used to adjust for the age of the patients and to determine if the results were still significant after accounting for the age differences.

## Results

There were 65 subjects in this study, with a total of 73 knees. After the final classification by the radiologist, there were 48 knees in the group with BME. There were a total of 15 knees or 31.125% in this group that had a TKA. There were 34 knees with a focal pattern of BME, 12 with a global pattern, and two with a cystic pattern. Of those with a focal pattern, 20.5% [7] had a TKA (Fig. 1). For those with a global pattern, 58.3% [7] had a TKA (Figs. 3 and 4), and for those with a cystic pattern, 50% [1] had a TKA (Fig. 6). These results are detailed in Table 1.

**Table 1:** Patterns of edema and percentage of those who subsequently received TKA

Pattern of edema	Total number	Number with TKA	Percentage with TKA (%)
Focal	34	7	20.5
Global	12	7	58.3
Cyst	2	1	50
All patterns of edema	48	15	31.2
None	25	2	8

**Fig. 7:**
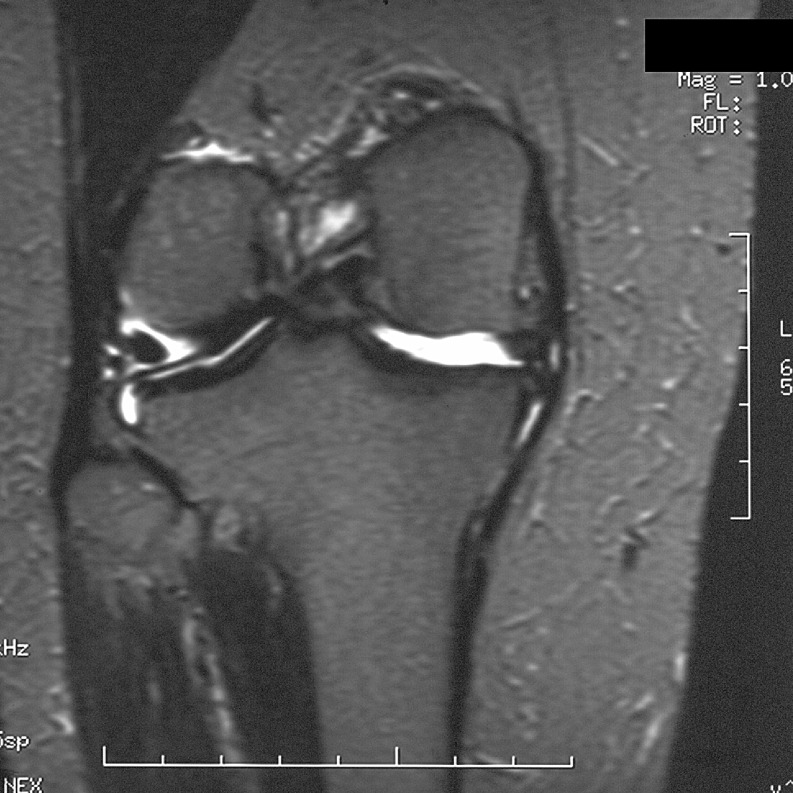
No bone marrow edema with a total joint replacement in a 66-year-old female patient. Coronal fast fat-suppressed T2-weighted image (TR 2500/TE 80/256 × 256) shows almost complete loss of hyaline cartilage in the medial compartment and an extruded, degenerative torn medial meniscus. Note the absence of marrow edema

There were 25 knees in the group with no BME. There were a total of two knees or 8% of those without BME who had a TKA (Fig. 7; see Table 1).

For statistical purposes, secondary to the small numbers of subjects in each subgroup, subjects were classified using the modified Noyes scale (0, 1, 2a, 2b, 3a, and 3b), and the data was grouped as cartilage damage less than 50% (0, 1, 2a) and cartilage damage greater than or equal to 50% (2b, 3a, 3b). For all three compartments of the knee, there were no statistical differences in rates of TKA between the groups that had less than 50% damage and the groups that had greater than 50% damage (see Table 2).

**Table 2:** Cartilage loss seen on MR, as graded by the modified Noyes scale, and its relationship to the occurrence of TKA

Variable	No TKA	Yes TKA	OR	95% CI	*p* Value
Medial cartilage loss <50%	17 (30%)	2 (12%)	2.77	0.71–10.72	0.14
Medial cartilage loss ≥50%	39 (70%)	15 (88%)			
Lateral cartilage loss <50%	42 (75%)	10 (59%)	2.06	0.74–5.70	0.16
Lateral cartilage loss ≥50%	14 (25%)	7 (41%)			
PF cartilage loss <50%	15 (28%)	3 (19%)	1.68	0.45–6.29	0.44
PF cartilage loss ≥50%	38 (72%)	13 (81%)			

For statistical analysis of the radiographs using the Kellgren–Lawrence scale, subjects who had scores of 0–2 (none, doubtful, or minimal) were compared with subjects who had a score of 3–4 (moderate, severe) to assess for statistical difference in the occurrence rate of TKA. Those subjects who had a Kellgren–Lawrence score of 3–4 were no more likely to have a TKA performed than those who had a score of 0–2 (see Table 3).

**Table 3:** Radiographic findings, as graded by the Kellgren–Lawrence scale, and their relationship to occurrence of TKA

Score	No knee replacement(*N* = 35)	Knee replacement(*N* = 11)	OR	95% CI	*p* Value
0–2	8 (23%)	1 (9%)	1.0006	0.9996–1.0016	0.23
3–4	27 (77%)	10 (91%)			

Table 4 shows the results of the subjects with BME, using the patterns of edema, compared to those without BME. Subjects who had BME of any pattern type were 5.48 times as likely to have a TKA when compared to subjects with no BME (*p* = 0.05). Upon further investigation, subjects with a global pattern of BME were 7.63 times as likely to have a TKA compared to subjects with focal, cyst, or no BME (*p* = 0.004). Subjects with global BME were 15.21 times as likely to get a TKA when compared to subjects without BME (*p* < 0.001).

**Table 4:** Comparison of bone marrow edema patterns and their relationship to occurrence of TKA

Variable	No TKA	Yes TKA	OR	95% CI	*p* Value
No BME	23 (41%)	2 (12%)	5.48	0.98–30.68	0.05
BME of any pattern	33 (59%)	15 (88%)			
Global BME	5 (9%)	7 (41%)	7.63	1.89–28.34	0.004
All other patterns (focal, cyst, none)	51 (91%)	10 (59%)			
Global BME	5 (18%)	7 (78%)	15.21	2.38–97.10	<0.001
No BME	23 (82%)	2 (22%)			

First, no bone marrow edema is compared with BME of any pattern. Next global BME is compared to all other patterns, including no edema. Finally, global BME is compared with no BME.

Table 5 demonstrates the results of a univariate comparison between subjects with BME and without BME, and their scores on the modified Noyes and the Kellgren–Lawrence classifications. For the medial compartment only, subjects who had high scores on the Noyes and Kellgren–Lawrence scales tended to have BME, whereas those subjects with lower scores on the Noyes and Kellgren–Lawrence scales tended to have no BME.

**Table 5:** Univariate comparisons between those knees with BME and those without BME (*t* tests)

Variable	Score	No BME	BME	*p* Value
Modified Noyes score lateral compartment	0–2a	20 (80%)	32 (67%)	0.232
	2b–3b	5 (20%)	16 (33%)	
Modified Noyes score medial compartment	0–2a	11 (44%)	8 (17%)	0.012
	2b–3b	14 (56%)	40 (83%)	
Modified Noyes score patellofemoral compartment	0–2a	4 (17%)	14 (31%)	0.193
	2b–3b	20 (83%)	31 (69%)	
Kellgren–Lawrence score lateral compartment	0–2	13 (100%)	26 (79%)	0.071
	3–4	0 (0%)	7 (21%)	
Kellgren–Lawrence score medial compartment	0–2	9 (69%)	11 (33%)	0.027
	3–4	4 (31%)	22 (67%)	
Kellgren–Lawrence score patellofemoral compartment	0–2	5 (42%)	11 (33%)	0.606
	3–4	7 (58%)	22 (67%)	

The presence of a meniscal tear did not correlate with subsequent TKA. There was no significant difference in the gender between the group that received a TKA and those that did not. The group of subjects who had a TKA were 12.6 years older than those who did not have a TKA (*p* = <0.001). In this study, the odds of having a TKA increase by about 1.11 times for each year increase in age.

A multivariable logistic regression model using generalized estimation was performed to account for the age difference in the groups and assess whether or not the results were still significant despite the age difference. Table 6 displays these results and demonstrates that, after accounting for the age difference, subjects who had BME of any pattern type were 8.95 times as likely to have a TKA when compared to subjects with no BME (*p* = 0.016). Subjects with global BME were 13.04 times as likely to get a TKA when compared to subjects without BME (*p* < 0.01). Subjects with a global pattern of BME were 5.45 times as likely to have a TKA compared to subjects with focal, cyst, or no BME (*p* < 0.05).

**Table 6:** Results of the multivariable logistic regression model using generalized estimation to account for the age difference in the groups that received a TKA versus those that did not

	Outcome: TKA			
	Variable	OR	95% CI	p-value
Model 1	BME versus no BMEf	8.95	1.49–53.68	0.0164
	Age	1.13	1.07–1.20	<0.0001
Model 2	Global BME versus No BME	13.04	2.06–82.58	0.0064
	Age	1.06	0.99–1.15	0.11
Model 3	Global BME versus all other patterns (focal, cyst, and no edema)	5.45	1.02–28.96	0.0467
	Age	1.107	1.05–1.17	0.0002

## Discussion

BME has previously been noted in the musculoskeletal system in osteoarthrosis and other conditions [1, 4–11, 13, 14, 20]. Felson and colleagues have examined BME and its relation to progression of knee osteoarthrosis and noted that BME is a potent risk factor for structural deterioration in knee osteoarthrosis [1, 7, 17]. Its relation to progression is explained in part by its association with limb alignment. In two of his studies, medial bone marrow lesions were seen mostly in patients with varus limbs and lateral lesions were seen mostly in those with valgus limbs [7, 17].

Link and colleagues [4] reviewed MR findings in osteoarthrosis and noted that cartilage lesions, BME patterns, and meniscal and ligamentous lesions were frequently demonstrated as MR changes in patients with advanced osteoarthrosis. However, in this study, there was no significant correlation between MR and clinical findings.

A recent study conducted by Kornaat et al. [21] examined multiple imaging findings and their association with clinical symptoms. Their results suggest that only findings of a large-joint effusion or the presence of an osteophyte in the patellofemoral compartment were associated with pain and/or stiffness. They found no association between BME and symptoms of pain or stiffness.

Three studies have reviewed the MR appearances with histopathology findings [6, 9, 10]. Bergman and colleagues in their study found that subchondral bone marrow changes were present in seven of nine patients undergoing total knee replacement [10]. Histopathologically, those regions showed focal areas where fibrous tissue replaced fatty marrow in the subchondral trabecular space. Zanetti and colleagues reviewed the histopathological findings in 16 patients who had MR before total knee replacement. The BME pattern consisted of normal tissue and a smaller proportion of several abnormalities including bone marrow necrosis, bone marrow fibrosis, abnormal trabeculae, BME, and bone marrow bleeding. They concluded that a BME pattern in knees with osteoarthrosis represents a number of non-characteristic histological abnormalities [9].

Nolte–Ernstein and colleagues examined the correlation between MR and histological findings of degenerative bone marrow lesions in experimental osteoartrhosis models in canine knee joints [6]. In these experimental lesions, the histopathology revealed 21 osteosclerotic lesions and 5 intraosseous cysts. Histopathological findings showed different degrees of osteosclerosis associated with bone marrow degeneration. Cystic lesions were of two types: subchondral epiphyseal cysts and synovial cysts within a large tibial osteophyte. High signal intensity on T2-weighted images and decreased signal intensity on T1-weighted images indicated high fluid content.

None of these prior studies specifically looked at the BME pattern on MR. Our classification of the patterns into global, focal, cystic, and absence of edema is an attempt to subdivide the presence or absence of edema in osteoarthrosis. However, this attempt is limited by the absence of histopathological findings.

We were surprised by the significantly increased risk of knee joint replacement with the global pattern of BME in relation to the other patterns. It appears that the more extensive and intense the BME, the more likely it is for the patient to have symptoms. The global pattern of BME was the best predictor of risk of TKA within 3 years, as those subjects with the global pattern were over five times as likely to receive a TKA when compared to those with any of the other patterns and over 13 times as likely to have a TKA when compared with subjects with no BME, after accounting for the age difference.

We were also surprised by the lack of association between cartilage loss and the likelihood of total knee replacement. Intuitively, one would think that the greater the cartilage loss, the more likely the possibility of total knee replacement. However, Link and colleagues [4] noted that clinical findings showed no significant correlation with the extent of cartilage loss on MR imaging. In this study, the lowest scores for pain and function loss were found in patients without cartilage lesions. However, the highest scores for pain, stiffness, and function loss were found in patients with less than 50% cartilage loss. The lowest scores for stiffness were found in patients with more than 50% cartilage loss and full-thickness lesions. These findings further support the theory that cartilage loss may not be the primary source of pain in patients with OA of the knee.

We did find, in the medial compartment only, that subjects with high grades of cartilage loss or advanced degenerative changes on radiographs tended to have some degree of BME. The lack of association between cartilage loss and the likelihood of TKA in our study could be a reflection of the limitations of the Noyes classification system. However, other investigators have also found that radiographic signs of cartilage loss may not relate to the degree of clinical symptoms. As mentioned above, Link and colleagues found no correlation with the extent of cartilage loss on MR and clinical findings. In their study, they also used a modified Noyes classification system [4]. Kornaat et al. also found no correlation between the extent of cartilage damage seen on MR imaging and clinical symptoms of pain and stiffness. They used a grading scale based on the maximum diameter of cartilage defects and the depth of the lesion based on the percentage of cartilage loss [21].

In clinical practice, OA is associated with the radiographic findings of joint space loss and subchondral sclerosis, presumably secondary to cartilage loss that results in pain. However, as stated earlier, there are no pain fibers in hyaline cartilage [3]. Furthermore, many patients have pain out of proportion to their radiographic findings. Our study may provide an insight into another possible mechanism for pain production in OA of the knee. While not every patient with OA warrants an MR scan, those patients without the classic presentation may benefit.

We did find that patients who had a TKA were 12.6 years older than those who did not have a TKA. Patients who are older are more likely to have OA and, in particular, more severe OA. Surgical replacement of the knee is more likely to occur in an older patient with OA than a younger patient with the same severity of OA. After further statistical analysis, we however found that our BME results were still significant despite the differences in age.

This paper has several limitations. Most patients who have a TKA do not get a pre-operative MR, and therefore, we may be dealing with a pre-selected population. What we have called “BME” on MR may not be true edema but may relate to other histologic findings as noted earlier. Our numbers are also small, and we had to group some of our subsets together for statistical analysis. Only one musculoskeletal radiologist was involved in the review of the radiology. There was also no arthroscopic correlation for cartilage defects or presence of meniscal tears. Our study is limited by the fact that it is retrospective. To generate a group of MR scans to review, we had to use a keyword search to identify potential subjects. We would have obviously missed all scans where the specific terminology “bone edema” or knee “osteoarthrosis/osteoarthritis” was not utilized. However, we feel that this is a relatively minor limitation, as we were able to generate 381 cases for review. Another potential limitation in the retrospective study design is the possibility of missing subjects who received follow-up at a different institution or those whose symptoms resolved without treatment.

In summary, we reviewed a series of knees in patients with osteoarthrosis and evaluated the pattern of BME, cartilage loss, radiographic findings when available, and the incidence of total knee joint replacement within a 3-year follow-up. Subjects with any bone marrow edema pattern were more likely to have a total knee joint replacement compared to subjects with no bone marrow edema. The worst prognostic pattern was the global pattern of bone marrow edema. Subjects who had a total knee replacement were also older than those who did not. However, even allowing for age, the global pattern of BME remained the variable with the highest statistically significant association with the incidence of total knee replacement.
